# 230. Risk factors for bacteremia in patients receiving extracorporeal membrane oxygenation (ECMO) during the COVID-19 pandemic

**DOI:** 10.1093/ofid/ofad500.303

**Published:** 2023-11-27

**Authors:** Curtis Hendrix, Christopher Radcliffe, Hossam Tantawy, Scott C Roberts

**Affiliations:** Yale New Haven Hospital, New Haven, Connecticut; Massachusetts General Hospital, Boston, Massachusetts; Yale New Haven Hospital, New Haven, Connecticut; Yale School of Medicine, New Haven, Connecticut

## Abstract

**Background:**

Patients receiving extracorporeal membrane oxygenation (ECMO) are at high risk for bacteremia which can cause substantial morbidity and mortality. We sought to evaluate risk factors for bacteremia in patients receiving ECMO during the COVID-19 pandemic to better characterize those most at risk and areas for prevention.

**Methods:**

A retrospective case control study evaluating patients receiving ECMO support at Yale New Haven Hospital from April 2020 – September 2021 was performed. Cases of patients who developed bacteremia were matched 1:2 to control patients on ECMO who had blood cultures drawn but who did not develop bacteremia. There were no set criteria for drawing the blood cultures; when to draw blood cultures were per provider’s discretion. Only the first set of blood cultures drawn were analyzed.

**Results:**

60 patients received ECMO support and had blood cultures drawn, 20 (33.3%) with bacteremia matched to 40 (66.7%) without bacteremia. Independent risk factors for bacteremia included being diagnosed with COVID-19 (70.0% vs 40.0%, p = 0.028), days on ECMO until culture (13.4 vs 6.2 days, p = 0.005), days on mechanical ventilation until culture (17.5 vs 7.7 days, p = 0.002), corticosteroid use (75.0% vs 40.0%, p = 0.011), ECMO type (80.0% veno-venous [VV] and 20.0% veno-arterial vs 37.5% veno-venous and 62.5% veno-arterial, p = 0.002), proning (60.0% vs 22.5%, p = 0.004), and preceding antibiotic days during the admission (16.1 days vs 9.3 days, p = 0.025). There was no difference in any evaluated infectious metric (white blood cell count, daily maximum temperature, procalcitonin, CRP, d-dimer, ferritin, fibrinogen, LDH) on the day of blood culture in those with compared to those without bacteremia.

**Conclusion:**

Patients receiving ECMO support who developed bacteremia were more likely to be on VV-ECMO, proned, receive corticosteroids, and be on mechanical ventilation for longer periods of time, all factors associated with severe COVID-19. Additional risk factors included more days on ECMO and prior antibiotic use. No laboratory metric was predictive of bacteremia, highlighting the challenges in accurately predicting bacteremia in the ECMO patient population.

**Disclosures:**

**All Authors**: No reported disclosures

